# Quantifying the Learning Curve in Ultrasound-Guided Vascular Access: Proficiency Metrics of Self-Taught Axillary Vein Puncture for CIED Implantation

**DOI:** 10.3390/medsci14010115

**Published:** 2026-02-27

**Authors:** Dimitrios A. Vrachatis, Konstantinos A. Papathanasiou, Ioannis Anagnostopoulos, Sotiria G. Giotaki, Maria Kousta, Christos Karavasilis, Christos Piperis, Panagiotis Tolios, Andreas Kaoukis, Konstantinos Raisakis, Georgios Giannopoulos, Theodore G. Papaioannou, Gerasimos Siasos, Spyridon Deftereos

**Affiliations:** 1 Department of Biomedical Engineering, National and Kapodistrian University of Athens, 11527 Athens, Greece; 2 Electrophysiology and Pacing Department, Eugenideio Clinic, National and Kapodistrian University of Athens, 11528 Athens, Greece; 3 Department of Cardiology, “G. Gennimatas” General Hospital of Athens, 11527 Athens, Greece; 4 Third Department of Cardiology, Aristotle University of Thessaloniki, ‘’Hippokration’’ General Hospital, 54124 Thessaloniki, Greece; 5 Third Department of Cardiology, National and Kapodistrian University of Athens, Medical School, Sotiria Chest Disease Hospital, 11527 Athens, Greece

**Keywords:** ultrasound-guided axillary vein puncture, self-taught experience, cardiac implantable electronic device, success rate, complications

## Abstract

Background: Ultrasound (US)-guided axillary vein puncture (AVP) is an established technique for cardiac implantable electronic device (CIED) implantation. Yet real-world data concerning shifting from conventional venous access into US-guided AVP are not widely available. Methods: This is a single-center prospective registry reporting safety (complications) and efficacy (success rate: i.e., accomplishment of the vein access utilizing only the initially employed approach) of self-taught US-guided AVP integration into the standard workflow of CIED procedures. Results: A total of 539 patients (mean age 71.5 ± 12.4 years old, 78.7% males) were treated in our institution over a three-year period. Regarding CIED type and lead number, 58.3% used an implantable cardioverter defibrillator, 32% used permanent pacemakers, and two leads were involved in 65.8% of the cases and three leads in 8.9%. Before integration of US-guided AVP, the venous access success rate was 93.5%. The US-guided AVP success rate was 377/400 procedures (94.2%). After the first semester of US-guided AVP utilization, a pattern of increased success rate was observed (*p* = 0.002) and remained stable over the following semesters. No major complication (periprocedural or 30-day mortality, hemothorax, pneumothorax and tamponade) occurred after US AVP integration in our workflow. Conclusions: The integration of US-guided AVP in a self-taught manner is feasible among electrophysiologists with experience in US-guided vascular access. A high success rate can be reached quickly and safely.

## 1. Introduction

Subclavian vein puncture (SVP), axillary vein puncture (AVP) and cephalic vein cutdown (CVC) are the main venous access techniques employed during cardiac implantable electronic device (CIED) implantation, yet they differ in terms of procedural success and complications [1]. AVP is an established method for lead insertion featuring low complication rates and high efficacy [2]. As compared to CVC, AVP has been associated with shorter procedural time, improved procedural success [3], reduced need for alternative venous access [4], and potentially less pain [5].

Ultrasound (US)-guided (vs. the anatomical landmark approach) AVP has the additional benefits of direct visualization of the vein and depth perception. Another advantage is the assessment of axillary vein size and/or occlusion, which can be impactful during CIED upgrade procedures, obviating the need for unnecessary incisions or venograms [6]. Further, US-guided AVP does not strictly require fluoroscopic confirmation of wire position; thus, the radiation exposure could be drastically reduced [7]. The risk of contrast-induced nephropathy during left ventricular lead placement increases substantially with the administration of large volumes of contrast. Minimizing the amount of contrast administration has been associated with reduced risk of kidney injury and reduced fluoroscopic time without compromising procedural success (left ventricular lead implantation) [8]. Thus, implanters should opt for contrast-free techniques among patients with impaired renal function and/or relevant allergies.

We describe our initial experience shifting our practice from conventional vein access to US-guided AVP in a real-world and self-taught setting and report on success rates and periprocedural complications.

## 2. Materials and Methods

### 2.1. Study Design

This is a single-center prospective registry of patients with an indication to CIED procedure between January 2023 and December 2025. All consecutive patients undergoing CIED implantations, revisions or upgrades were analyzed. US-guided AVP was established from December 2023 onwards and was performed by electrophysiologists with no prior application of the technique, yet experienced in US-guided femoral access, by using a standard vascular US probe and minimal modification to the workflow.

This study was conducted in accordance with the Declaration of Helsinki, and the protocol was approved by the Ethics Committee of Eugenideio Clinic, National and Kapodistrian University of Athens (PN01) on 9 January 2023.

### 2.2. Procedure

Procedures were performed in the electrophysiology laboratory of a referral hospital by three experienced electrophysiologists (DAV, IA, MK), often with a fellow participating. The access site was at the discretion of the operator. Perioperative management of anticoagulants and antiplatelet drugs was aligned with the international consensus [9] and antibiotic prophylaxis was given one hour before the incision. All patients were prepped in the clavicular area and draped in the standard sterile fashion, and 1% lidocaine was infiltrated parallel to the deltopectoral groove before AVP. Intravenous fluids were administrated to increase the axillary vein diameter and the operating table was placed in the Trendelenburg position by tilting it head-down by ~15°.

**Figure 1:**
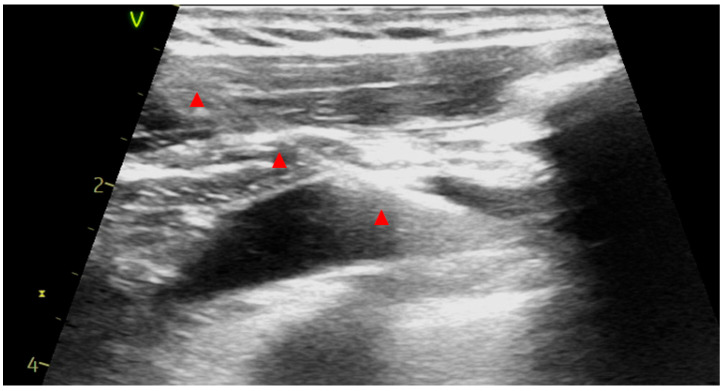
Long-axis (in plane) view of axillary vein after insertion of a J-tip guide wire; note the ability to visualize the full course of the wire in this view (red marks).

A portable laptop US system was utilized and a standard linear US probe was prepared with non-sterile US gel and covered by a long sterile plastic sheath. The probe prepped with sterile US gel and with the marker oriented cranially was held by the non-dominant hand and maneuvered subtly in the infraclavicular region until the axillary vein and artery could be visualized in long-axis (in-plane) and short-axis (cross-sectional) views. In order to identify the course of the axillary artery, the probe was tilted superiorly (to identify the axillary artery) and then inferiorly to finally locate the axillary vein. Thin walls, superficial position, cephalic vein confluence, inspiratory collapse, lack of pulsation and complete compressibility along with occasional use of color Doppler and pulse wave velocity analysis were used to differentiate artery from vein. An 18-gauge needle was employed (a 10 mL syringe was attached or not according to operator’s preference) and advanced under continuous US guidance over the long-axis view until the needle tip approached the vein wall; at this point short jabs were usually needed to enter the axillary vein lumen (Figure 1). Vascular access was confirmed by the produced blood backflow. Subsequently, a 0.035-inch J-tipped guide wire was advanced into the axillary vein. Afterwards, short-axis view was utilized to confirm vein cannulation. Then, the J-tip guide wire was advanced under fluoroscopy to the central venous system. The process was repeated for each lead needing additional vascular access, especially if three leads were implanted or the retained guide wire technique was utilized according to the implanter’s preferences. When vein access was deemed successful, an incision was made and the J wire was meticulously freed from the subcutaneous tissue and its distal part was pulled through the incision. The rest of the procedure followed the standard practice. Absorbable sutures were utilized for subcutaneous and intradermic closure.

CIED procedures without US guidance were performed according to conventional implantation techniques [9]. Standard measures to keep radiation exposure as low as reasonably achievable (ALARA principles) were followed, including low frame rates, collimation, minimal magnification, short fluoroscopy instead of cine, and reduced distance between the patient and the detector.

All patients were evaluated with a post-procedural chest X-ray and were discharged the following day. Routine follow-up for clinical assessment (including incision inspection, complication detection and CIED checks) was scheduled one week and one month after discharge.

The study protocol adhered to the ethical guidelines of the Declaration of Helsinki and all patients provided their informed consent for CIED implantation.

### 2.3. Outcomes

Major access-related periprocedural complications (periprocedural and 30-day mortality, hemothorax, tamponade, and pneumothorax) were evaluated over the 30-day period and unsuccessful US-guided AVP attempts were collected for each procedure. Efficacy was defined by procedural success (accomplishment of the vein access utilizing only the initially employed approach, e.g., US AVP or CVC or fluoroscopic AVP or SVP).

### 2.4. Statistical Analysis

The Kolmogorov Smirnov test was used to test for normality. Continuous variables are presented as means ± standard deviation (SD). Categorical variables are presented as relative frequencies and comparisons between groups were made using the Pearson chi-square test for categorical variables. A *p*-value < 0.05 was considered statistically significant. IBM SPSS Statistics for Windows, version 28 (IBM Corp., Armonk, NY, USA) was used.

## 3. Results

A total of 539 patients were treated in our institution over the study period. The procedures were roughly equally distributed between the operators. The majority of the patients were males (n = 424, 78.7%) with a mean age of 71.5 ± 12.4 years old. A share of 98.8% (533/539) of the CIEDs under evaluation were magnetic resonance imaging (MRI) conditional. First CIED implantation comprised the majority of the procedures 87.8% (n = 473), while the remaining 66 (12.2%) cases were revisions, replacements, upgrades or extractions. As far as the type of CIED is concerned, 58.3% (n = 314) were implantable cardioverter defibrillators (ICDs), 32% (n = 173) permanent pacemakers (PPMs), 8.9% (n = 48) cardiac resynchronization therapy defibrillators (CRTDs), and 0.74% (n = 4) left bundle branch pacing (LBBP). A total of 2 leads were involved in 65.8% (n = 355) of the cases, 1 lead in 25.2% (n = 136) and 3 leads in 8.9% (n = 48).

A total of 84 cases (16%) in our registry were performed before US AVP establishment and 455 (84%) after. After shifting into US AVP, the type of procedure did not differ (Pearson chi-square *p* = 0.77) as shown in Table 1.

After excluding 44 cases (extractions, replacement and/or revision cases), 495 access sites were analyzed in total. A share of 75% were left US AVP (n = 371), 1.6% right US AVP (n = 8), 11.9% fluoroscopically guided AVP (n = 59), 9.3% left CVC (n = 46), 0.6% right CVC (n = 3) and 1.6% SVP (n = 8). As expected, the type of access site differed significantly before and after establishment of US-guided AVP (*p* < 0.001), and it is provided in Table 2.

**Figure 2:**
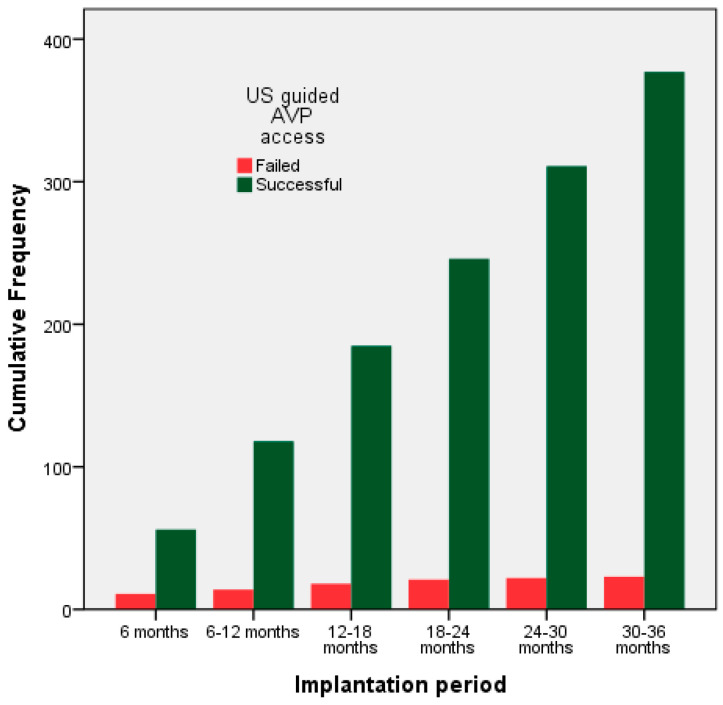
Successful (green columns) and failed (red) attempts of ultrasound-guided axillary vein puncture over the study period per semester.

Before shifting into US-guided AVP, the venous access success rate was 93.5% (73/78). In five cases, an alternative venous access was needed to complete the procedure. Among 417 patients after US-guided AVP establishment, 17 CIED procedures were carried out without US guidance based on operator’s preference, mainly due to time constraints and US system availability. The US-guided AVP success rate was 377/400 procedures (94.2%). Alternate sites after failed US AVP were as follows: 52% CVC (n = 12), 39% SVP (n = 9), 4.5% right-sided US AVP (n = 1), and 4.5% right-sided fluoroscopically guided AVP (n = 1). After dividing implantation period into 6-month intervals, a pattern of increased success rate was observed (*p* = 0.002) and stabilized after the first semester as shown in Figure 2 and Table 3.

As far as major complications are concerned, no deaths were encountered (periprocedural and 30-day mortality), nor were tamponade or hemothorax observed. Prior to US AVP establishment, two patients developed pneumothorax (one of them needed chest tube insertion), while afterwards no such cases occurred.

## 4. Discussion

The present study represents a real-world registry confirming that self-taught US-guided AVP is feasible, effective and safe. A high success rate may be reached within 6 months after a relatively small number of CIED implantations (approximately 65 cases).

In a case–control study by Bhuva AN et al., the success rate of US-guided AVP after skin incision (our study entailed US-guided AVP before skin incision) was 97% with no periprocedural complications and an acceptable learning curve (needle to wire time) which improved over time, after approximately 45 cases [7]. In our registry, the success rate stabilized to 95% after 6 months, which translated into roughly 65 cases. Nikorowitsch J et al. analyzed data from 986 patients and reported that US-guided AVP has a steep learning curve during the first 25 cases, which is comparable to our data (approximately 22 cases per operator), and complications are rare (0.3%) [10]. Another study found that the US-guided AVP success rate is 70% during the initial self- learning phase [11]. In our study the initial success rate was higher at 84% and could be attributed to previous experience in US-guided vascular access and the larger number of cases analyzed during the training phase (67 over 6 months vs. 46 over 3 months by Liccardo et al.).

The aforementioned advantages of US-guided AVP have been confirmed by randomized clinical trials (RCTs) and meta-analyses. In an RCT, US-guided AVP had a high success rate similar to that in our findings (94%) and complication rates were comparable with those of conventional venous access techniques. Among inexperienced implanters, access time improved significantly over time [12]. A patient-level meta-analysis of 700 patients indicated that US-guided AVP is associated with less radiation exposure, shorter time to venous access and a lower number of attempts to venous access as compared to fluoroscopy-guided AVP [13]. Radiation exposure during ablation and CIED procedures can be drastically reduced with the implementation of non-fluoroscopic approaches [14]. Zero to minimal fluoroscopy strategies (either three-dimensional electroanatomic mapping systems or transthoracic echocardiography) during CIED implantation have been proven safe and effective [15], yet more randomized data are needed. The integration of US-guided AVP during non-fluoroscopic CIED procedures should be further investigated in terms of procedural safety and efficiency since it is an established method to curtail radiation exposure and contrast use.

Perna et al. have recently published their experience using a microintroducer kit for US-guided AVP among 433 patients. The technique was safe and effective, and reduced bailout venograms and fluoroscopy times [16]. An axillary vein diameter above 6 mm has been shown to be predictive of successful cannulation [17]. The Valsalva maneuver might be useful in cases of inadequate axillary vein size, leading to an average 5 mm diameter increase in 81% of the patients [18]. Although US-guided AVP could be advantageous among obese patients, it should be underlined that increased body mass index [19] and increased vein depth [20] have been associated with axillary vein cannulation failure [21]. The intra-pocket technique combined with a dedicated US probe with a smaller footprint might mitigate these anatomical limitations and has been examined in both randomized [22] and observational studies [23,24,25].

The advent of US-guided AVP should not create the misconception that alternate venous access sites and techniques are of little importance. In a recently published RCT, the axillary vein was not adequately visualized in 20% of the cases and crossover to conventional techniques was needed [26]. Under these circumstances of suboptimal US imaging of vascular structures, the utilization of minimal amounts (<55 mL) of contrast combined with short duration fluoroscopy could be clinically impactful, especially during complex CIED upgrade procedures and CRTD implantation [8]. Thus, implanters should remain proficient with CVC and SVP, especially in light of anatomic variations and CIED upgrade procedures.

### Limitations

Our study has a series of limitations. First, this is a single-center registry involving a relatively low number of CIED procedures annually; thus, the US-guided AVP learning curve could be reached in a shorter time period among higher-volume centers. Nevertheless, basic experience in US-guided vascular access should not be neglected. Second, the time needed to obtain venous access was not available for comparative analysis. However venous access time varies among studies and it depends on the US probe footprint, body mass index, skin incision before or after AVP, and the type of puncturing needle (micropuncture 21-gauge versus conventional 18-gauge needle) [23]. Lastly, long-term follow-up is needed to assess late complications.

## 5. Conclusions

Shifting from conventional venous access into US-guided AVP in a self-taught manner may be feasible among electrophysiologists with experience in US-guided vascular access. A high success rate can be reached shortly without significant complications.

## Figures and Tables

**Table 1:** Type of procedure before and after establishment of ultrasound-guided axillary vein puncture.

Procedure Type	Before (n = 84)	After (n = 455)
New Implantation	73	400
Upgrade	4	12
Extraction, Revision or Replacement	7	43

**Table 2:** Type of venous access site before and after establishment of ultrasound-guided axillary vein puncture.

Venous Access Site	Before (n = 78)	After (n = 417)
Left CVC	25 (32%)	21 (5%)
Right CVC	3 (3.8%)	0
Subclavian vein puncture	4 (5.1%)	4 (1%)
Fluoroscopically guided AVP	46 (59%)	13 (3.1%)
Left US-guided AVP	0	371 (89%)
Right US-guided AVP	0	8 (1.9%)

AVP: axillary vein puncture, CVC: cephalic vein cutdown, US: ultrasound.

**Table 3:** Success rate for ultrasound-guided axillary vein puncture over the study period per semester.

Implantation Period	6 mo	6–12 mo	12–18 mo	18–24 mo	24–30 mo	30–36 mo
Success rate (%)	56/67 (84%)	62/65 (95%)	67/71 (94%)	61/64 (95%)	65/66 (98%)	66/67 (98%)

mo: months.

## Data Availability

The original contributions presented in this study are included in the article. Further inquiries can be directed to the corresponding author.

## Abbreviations

The following abbreviations are used in this manuscript:

ALARA	as low as reasonably achievable
AVP	axillary vein puncture
CIED	cardiac implantable electronic device
CRTD	cardiac resynchronization therapy device
CVC	cephalic vein cutdown
ICD	implantable cardioverter defibrillator
PPM	permanent pacemaker
SVP	subclavian vein puncture
US	ultrasound
